# Prevalence of Lyme Carditis in Patients with Atrioventricular Blocks

**DOI:** 10.3390/ijerph192214893

**Published:** 2022-11-12

**Authors:** Krzysztof A. Kaczmarek, Katarzyna Szwabe, Irmina Urbanek, Pawel Ptaszynski, Aleksander Strzelecki, Jerzy K. Wranicz, Iwona Cygankiewicz

**Affiliations:** 1 Department of Electrocardiology, Central University Hospital, Medical University of Lodz, 90-647 Lodz, Poland; 2 Department of Laboratory Diagnostics and Clinical Biochemistry, Medical University of Lodz, 90-647 Lodz, Poland

**Keywords:** atrioventricular block, *Borrelia burgdorferi*, Lyme carditis, pacemaker

## Abstract

Infections with *Borrelia* may cause cardiac conduction system abnormalities, including atrioventricular blocks (AVBs). Therefore, we aimed to identify patients in whom Lyme carditis (LC) could be considered as the initial diagnosis among consecutive subjects who were referred for implantation of a permanent pacemaker due to symptomatic AVBs. To date, such a systematic evaluation has not been reported yet. Validation of the Suspicious Index for Lyme Carditis (SILC) in our study population was considered as an additional goal. We investigated consecutive patients with AVB admitted to our department for a pacemaker implantation. Serological diagnostic tests against *Borrelia burgdorferi sensu lato* (Bbsl) were performed in those with no obvious cardiac causes of AVB. The final study population consisted of 130 patients (80 M, mean age 67.4 ± 17.6). Lyme carditis was assumed as the initial diagnosis in 16 patients (12%) based on ABV and IgM Bbsl seropositivity. The patients with LC were younger and more frequently manifested constitutional symptoms of infection and fluctuating AVB. The highest prognostic value for identification of LC patients was obtained for the modified SILC, which included the following parameters: (1) age lower than 75 years; (2) risky outdoor activity and living in the countryside; (3) tick bite; (4) constitutional symptoms of Lyme disease; (5) erythema migrans; (6) male sex and (7) fluctuating atrioventricular block. We concluded that diagnostics for LC should be routinely considered in patients with advanced AVB. Modified SILC may identify the patients at risk of LC.

## 1. Introduction

Cardiac bradyarrhythmias resulting from advanced atrioventricular conduction abnormalities are potentially life-threatening events; thus, after the exclusion of reversible causes, they usually lead to the implantation of a permanent pacemaker [1]. Infection-induced atrioventricular blocks (AVBs) have been recorded as case reports or series involving a limited number of patients.

*Borrelia burgdorferi* is increasingly noted as responsible for a considerable number of infection-related AVBs [2], and AVBs have been reported as the most common clinical presentation of LC (accounting for 80–90%) [3,4]. These blocks may potentially be resolved by an antibiotic therapy; therefore, the initial diagnosis of *Borrelia* infection may exert a significant impact on the therapeutic strategy in early pacemaker implantation.

Recently, a Canadian group of cardiologists introduced a score for clinical practice which could help to identify patients with AVB most probably attributed to the infection with *Borrelia* [5]. The score, called the Suspicious Index in Lyme Carditis (SILC), was constructed based on the analysis of previously published reports. SILC consisted of six parameters, and from 1 to 4 points were given for each of them (1 point for: male sex, outdoor activity/endemic area and age below 50 years; 2 points for constitutional symptoms of Lyme disease; 3 points for history of tick bite; and 4 points for revealing erythema migrans); the point total is the score. Low suspicion of LC was a score of 0–2 points, intermediate—3–6 points and high—for 7–12 points.

We aimed to identify patients in whom LC should be considered as the potential underlying cause of AV conduction disturbances by performing a systematic analysis for seroprevalence of *Borrelia burgdorferi sensu lato* (Bbsl) in consecutive individuals who were referred to the cardiology department for a permanent pacemaker implantation due to symptomatic AVB. Validation of SILC score in our study population was considered as an additional goal.

## 2. Materials and Methods

### 2.1. Study Group

**Figure 1:**
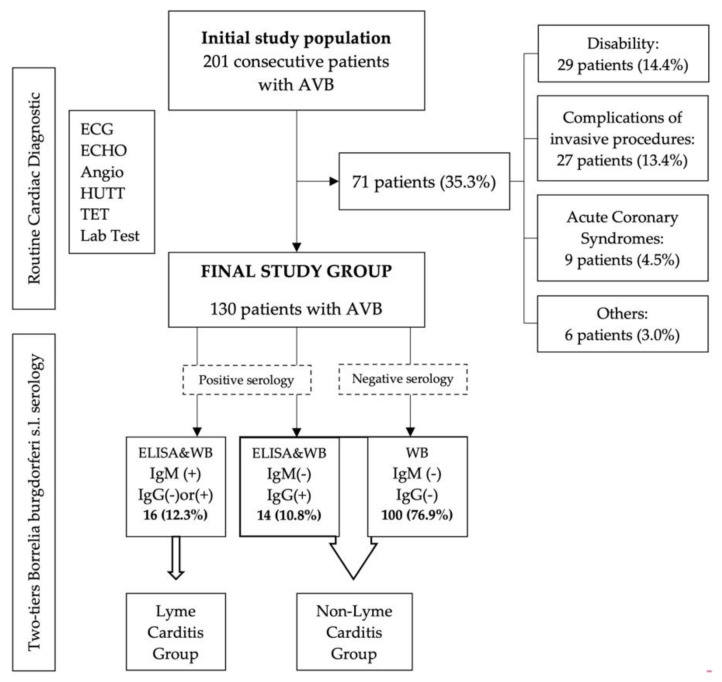
The workflow of the study. Figure legend. AVB—atrioventricular block; ECG—electrocardiogram, ECHO—echocardiography; Angio—coronary or/and pulmonary angiography; HUTT—head-up tilt test; TET—treadmill exercise test; Lab Test—laboratory tests; s.l.—sensu lato; ELISA—enzyme immunoassay test; WB—immunoblot test; IgM/IgG—immunoglobulin class M/G; (−) negative; (+) positive.

Initially, our study population included patients with AVB consecutively admitted to the Department of Electrocardiology Medical University of Lodz, Poland within a period of 12 months for the implantation of a permanent pacemaker. The exclusion criteria were as follows: (1) disabilities that restricted outdoor activities (at least for more than one year), which consequently eliminated a possibility of a recent tick bite; (2) secondary causes of conduction system dysfunction other than infections, such as antiarrhythmic drugs, metabolic disorders (dyselectrolitemia, hypothyroidism and others), acute coronary syndromes, acute pulmonary embolism and AVB that developed as complications of invasive cardiac procedures (percutaneous or open chest surgery). Eventually, the patients who did not meet the exclusion criteria constituted the final study population and had a serology test for infection with Bbsl performed. The individuals were then included in the further detailed analysis (Figure 1).

### 2.2. Diagnostic Tests

Cardiac diagnostic management, which is routinely executed in the course of bradyarrhythmia patients evaluation, was performed in all patients enrolled in the study. These diagnostic tests included resting surface ECG, prolonged ECG monitoring (bedside telemetry or 12-h Holter), echocardiography, as well as blood and urine tests. Additionally, exercise ECG testing, coronary angiography and/or head-up tilt test were performed when appropriate. The selection of tests was left to the cardiologist’s discretion and depended on clinical presentation of particular patients.

Two-tier serology method with initial enzyme immunoassay test (ELISA, Euroimmun AG, Lubeck, Germany) and immunoblot (EUROLINE Borrelia RN-AT adv IgM and Euroline Borrelia IgG, Euroimmune AG, Lubeck, Germany) as a confirmatory test were performed in all patients in the final study group (Figure 1). The enzyme immune assay was interpreted as negative (<16 RU/mL), borderline (≥16 and <22 RU/mL) or positive (≥22 RU/mL) following the manufacturer’s recommendations. All positive and borderline results were confirmed with immunoblot tests. As advanced AVBs are potentially life-threatening events, in some cases both tests were sent to be analyzed simultaneously to shorten the diagnostic process. The decision on a stepwise or simultaneous analysis was left to the discretion of a cardiologist. Depending on the serology results (Table 1) the patients were assigned to one of three groups: negative Bbsl serology (NBS), Lyme carditis (LC) or possible Bbsl previous exposure (pPBE). NBS was defined as negative results in both IgM and IgG antibodies in ELISA alone or altogether with confirmatory immunoblot test (if performed). Positive serology was determined if any or both classes of tested antibodies (IgM/IgG) produced positive results in the confirmatory immunoblot test. Possible previous exposure for Borrelia sp. group (pPBE) included patients with IgG seropositivity but IgM-negative according to western blot test. Lyme carditis occurs during the early disseminated stage, so usually by the time of evaluation there should be IgM seropositivity. Therefore, the initial diagnosis of LC was considered in patients with AVB in whom IgM antibodies were found positive in the confirmatory immunoblot test [6,7].

In clinical practice, patients with LC diagnosis at admission typically receive antibiotics, which seem to be highly effective in resolving advanced atrioventricular conduction abnormalities. Non-LC patients (both NBS and pPBE), however, are not recommended to be treated with antibiotics and usually have a permanent pacemaker implanted unless other reversible causes of AVB are found [1,2]. Therefore, based on the aforementioned different clinical management in LC vs. non-LC individuals, our analysis was focused on these two groups of patients (Figure 1).

### 2.3. Clinical Covariates

The patients who had Bbsl serology performed were analyzed in terms of demographic and clinical characteristics including typical cardiovascular and metabolic comorbidities (hypertension, ischemic heart disease, heart failure, atrial fibrillation, dyslipidemia, diabetes mellitus). Additionally, a detailed analysis of bradyarrhythmia was performed with special attention paid to bradycardia-related symptoms, the identification of the most advanced type of AVB and fluctuating degree of conduction abnormalities recorded on ECG (in standard or prolonged recordings) during the diagnostic process.

### 2.4. Risk of Lyme Carditis

The factors related to an increased possibility of a tick bite and/or infection with *Borrelia* were collected and analyzed. Based on previous papers, summarized by Besant et al. [5], the following parameters were deemed as related to such a risk: (1) a tick bite reported by a patient, (2) age below 50 years, (3) erythema migrans, (4) constitutional symptoms observed in borreliosis and (5) living or staying in endemic areas and/or risky outdoor activity. All the above listed parameters that form SILC were calculated for all patients in our population. SILC was analyzed as continuous and categorized values. The original results of SILC divided patients into three groups of LC risk (low, intermediate and high), but two of them (intermediate and high) were merged together and managed according to the identical pathway. Therefore, by following this clinical approach, we analyzed SILC as trichotomous and dichotomous categories, with the latter one dividing patients into lower and higher risk of LC. Modification of SILC tailored to our study population was planned (Table 2).

### 2.5. Statistical Analysis

Statistical analysis was performed using Statistica software (ver. 13, StatSoft Inc., Tulsa, OK, USA) and SPSS (IBM Corp., Armonk, NY, USA). Continuous and categorical variables are shown as mean ± standard deviation and frequencies, respectively. For comparison between groups, depending on data distribution, parametric (t-Student test and ANOVA analysis) or non-parametric (U-Mann Whitney test and Kruskal-Willis H test) statistics were applied accordingly. The chi-squared test and Fisher’s exact test were used to compare categorical data.

The diagnostic ability of LC identification with SILC and its modifications was estimated with receiver operating characteristic (ROC) analysis. The ROC curves were plotted for each SILC variant. The area under curve (AUC), asymptotic significance and cut-off values were calculated. These cut-off values served to divide the study group dichotomously into the higher and lower LC risk patients for subsequent logistic regression and odds ratios (OR) calculations (OR ± 95% coefficient interval).

For all analyses, the values of *p* < 0.05 were considered statistically significant.

### 2.6. Bioethical Committee Approval

The study was performed according to the Helsinki Declaration with Good Clinical Practice standards and was approved by the local bioethical committee (Nr RNN 194/20/KE). All the patients gave their informed consent for diagnostic and therapeutic procedures at admission to hospital.

## 3. Results

The initial study population included 201 consecutive patients, 71 of whom (35.3%) were excluded mainly due to outdoor activity-restricting disabilities, postprocedural conduction system injury or AVB complicating acute coronary syndromes (Figure 1).

Thus, the final study group involved in our further analysis consisted of 130 patients (80 men, 61.5%; mean age 67.4 ± 17.6 years) who underwent serological testing for Bbsl. The majority of these patients had arterial hypertension (84; 64.6%), approximately one fourth of them had ischemic heart disease (34; 26.2%) and one fifth suffered from diabetes (40; 19.9%) and dyslipidemia (44; 21.9%). Atrial fibrillation was reported by 23 patients (17.7%). One sixth (22; 16.9%) of the patients had a history of heart failure, including 13 individuals (10.0%) with a reduced and mildly reduced ejection fraction. Mean ejection fraction for the final study group was 58.5 ± 5.2%.

Positive serology, defined as positive IgM and/or IgG in confirmatory immunoblot test was found in a group of 30 patients (23.1%). IgM seropositivity indicating acute phase was found in 16 of them (12.3%), which together with clinical scenario of AVB led to initial diagnosis of LC. None of these patients had pericardial effusion, chest pain or other clinical or echocardiographic features of pericarditis. Similarly, we found neither signs nor symptoms of acute myocarditis or endocarditis in any of our patients. Therefore, the atrioventricular conduction disturbances were found to be the unique clinical presentation of LC in our study group. The remaining 100 patients (76.9%) had negative serology and together with 14 PBE patients (10.8%) constituted our non-Lyme carditis group (non-LC). The LC group accounted for 8.0% (16 patients out of 201 individuals) of the initial study population.

Demographic and clinical characteristics of the LC patients were similar to those of the rest of the group (Table 3). Lyme carditis patients were younger (61.9 ± 17.0 vs. 68.2 ± 17.6); however, this difference showed merely a trend toward statistical significance (*p* = 0.08). The symptoms attributable to bradycardia as well as the most advanced atrioventricular conduction abnormalities were similar in patients with LC and the remaining individuals (Table 3). Complete heart block was recorded in almost 40% of the patients (LC vs. non-LC: 37.5% vs. 39.4%; *p* = 0.89), and the second-degree AVB was identified in over half of patients in both groups (respectively: 71.5% vs. 53.5%, *p* = 0.68). The remaining patients (under 10%) had trifascicular block or first-degree AVB with recurrent syncopal symptoms. Fluctuating atrioventricular conduction abnormalities were observed significantly more often in the individuals from the LC group (75.0% vs. 49.9%; *p* = 0.04).

All the assessed components of the original SILC showed similar values in both groups (Table 3), with one exception i.e., constitutional symptoms which occurred more frequently in LC than in non-LC patients (12.5% vs. 0.9%; *p* = 0.04, respectively). SILC analyzed as continuous values was found significantly higher in LC as compared to non-LC patients (3.00 ± 2.42 vs. 2.01 ± 1.12, *p* = 0.03). Nevertheless, while analyzed as LC risk categories used to define high and low risk patients, it showed barely a trend toward significance in trichotomous analysis and lost its predictive value when analyzed as dichotomous clinical categories (high vs. low risk) (Table 4).

It has to be underscored that originally described SILC was constructed in North America where conditions differ from European ones in terms of ‘endemic area’ definition. Taking into account that 100% of our patients (inhabitants of central Poland) met a US- defined criterion of ‘living in endemic area’ we redefined ‘endemic area’ as ‘living in countryside’. Furthermore, based on the available literature [8,9,10], we also redefined ‘risky outdoor activity’ as specifically connected with certain professions or amateur occupations: environmental specialist (biologists, ecologists etc.), forest workers, hunters and others (Table 4). In our study population the ‘age < 50’ criterion used in the original SILC did not differ between LC and non-LC group. On the other hand, we noticed a trend toward older age (>75 years) in patients from non-LC group (*p* = 0.07) (Table 3). Fluctuating AVB, which is not considered in the original score, was more frequently observed in LC group of our population.

Taking into consideration the findings from our study and the abovementioned reasoning based on US vs. Central Europe difference, we tested the modifications of the original SILC score tailored to our studied population of patients with AVB routinely admitted to cardiology departments. Four modifications to the original SILC as shown in Table 2 were tested. They reflected a different definition of the endemic area, a different age cut-off value and the presence of fluctuating AVB.

The calculations of AUC performed for the original and modified SILCs showed that AUC larger than 0.7 (good classifiers) was obtained for SILC_2_, SILC_3_ and SILC_4_, (Table 5). The cut-off values of the original and modified SILC indices were used for logistic regression dichotomously categorizing patients into low- and high-risk groups. The original SILC showed only a trend toward significance (*p* = 0.054). The highest OR was found for a modified SILC which included the age < 75, living in countryside/risky outdoor activities, a tick bite, erythema migrans, male sex and the presence of fluctuating AVB (OR = 12.00 [2.56; 56.22], *p* = 0.0015). When the parameter of fluctuating AVB was added, it doubled the OR recorded for SILC 1, 2 and 3 modifications (Table 5).

## 4. Discussion

Our main finding indicates that LC should be considered as the initial diagnosis in a relatively high number of patients (8%) admitted with atrioventricular blocks for a permanent pacemaker implantation. The prevalence of LC was even higher (12%) in the patients without obvious causes of a cardiac conduction system dysfunction. Such an observation made in consecutive patients with AVB has not been previously published. Furthermore, we documented that the original SILC score can be modified so that it shows better diagnostic performance in European populations. This emphasizes the need for adjustment of the SILC component definition that determines the risk of exposure to tick bites to include regional differences in the concept of tick endemic areas. Finally, we confirmed that the fluctuating nature of AVBs reported previously in case reports should be taken into account as a strong predictor of LC in patients referred for a pacemaker implantation.

Lyme disease is the most common vector-borne illness in the northern hemisphere, including Europe. A general trend toward an increase of Lyme disease incidence in Europe has been observed [11,12]. In Poland, where our study was conducted, the number of Lyme disease detected cases has fluctuated between approximately 20,000 to 75,000 annually in the last decade [13]. It is estimated that cardiac involvement could be found in 1–4% of European patients with Lyme disease; however, systematically analyzed data in this field is lacking. The predominant LC presentation observed in 80–90% of patients usually includes the second- and third-degree atrioventricular blocks [3,4]. If this data is applied to Poland, approximately between 200 to 2500 Lyme disease patients could be expected to demonstrate advanced (>2nd degree) AVB. On the other hand, in Poland approximately 11,000 patients have permanent pacemakers implanted each year for AVBs [14]. According to our findings, 8% of them might have LC, which gives approximately 900 patients possibly suffering from *Borrelia* infection. The order of magnitude in both scenarios is similar, which suggests that the LC as a cause of AVBs should be routinely taken into consideration. However, according to a recent Danish study, LC is clearly underestimated as the etiology of AVB as it is diagnosed in only a small percentage of relatively young patients (0.9%) [15].

There are several reasons why we did not observe statistical significance for the original SILC score. Most importantly, this index was created based on case reports and/or small series of LC cases [5,16]. Obviously, it is more popular to publish case reports with favorable outcomes than therapeutical failures. Routinely, LC as the initial diagnosis is usually taken into account in younger subjects, who are more likely to be investigated more thoroughly prior to a pacemaker implantation. However, in clinical practice, patients consecutively admitted to a cardiology department for a pacemaker implantation are more frequently older than those described in case reports on LC. In the majority of elderly patients, the pathophysiology of AVBs is connected with age-related sclerofibrosis of the cardiac conduction system. On the other hand, AVB disclosure in younger patients ought to be alarming for other causes then senile degeneration of cardiac conduction system [17]. Nevertheless, the possibility of LC as the underlying cause has not been systematically studied in elderly patients with AVBs. We believe that the difference between the age cut-off value originally used in SILC and our modified age criterion is most likely driven by the inclusion of published cases and/or small patients’ series rather than the systematically recruited population in the original SILC. The other major reason which led us to elaborate on the modification of SILC is related to the Central European location of our study. The environmental conditions in Europe are different than in North America, firstly, because of different prevalence of different species of ticks and *Borrelia* bacteria [18]. Additionally, *Borrelia* pathogen-transmitting ticks invaded the whole territories of Central European countries including urban areas, which expanded the risk of tick bite practically to anybody who undertakes outdoor activity [13,19,20]. However, a high prevalence of seropositivity to Bbsl is known to affect people who spend a considerable amount of time outdoors due to their professional occupation [9,10]. This argument was the reason for the alteration of ‘endemic area/outdoor activity’ component in SILC in order to result in a more specific indication of the population at risk of a tick bite.

In our study, the fluctuation of AVB was more frequently observed in LC patients (75% vs. 49%, *p* = 0.04). This observation in patients admitted to hospital, prior to antibiotic therapy initiation as well as during such a therapy, was previously reported by other authors [21]. The inclusion of fluctuating AVB at admission as an additional parameter significantly improved the identification of LC patients. Therefore, we suggest that atrioventricular conduction behavior observed on ECG monitoring should be included in the diagnostic process of AVBs that could be potentially LC-related.

Several important limitations of our study should be acknowledged. Firstly, this research was a single-centered study, which might limit the extrapolative power of our findings. Additionally, our population was relatively sparse, which precluded us from performing more in-depth analyses. Furthermore, our data refer only to the initial diagnosis of LC. Nevertheless, it is the initial diagnosis that determines the therapeutical approach, including antibiotic treatment vs. early decision on pacemaker implantation. All these restrictions are a compelling reason for a careful interpretation of our conclusions. As our study revealed that a significant percentage of AVB patients have Borrelia seropositivity, there is a need for further investigation in this field. Multicenter larger clinical trials reflecting different endemic areas with long-term follow-up should be initiated.

## 5. Conclusions

Lyme carditis as the potential cause of AV conduction disturbances was diagnosed in a relatively high percentage (approximately 10%) of the patients admitted for a permanent pacemaker implantation due to AVB. Therefore, diagnostics of LC should be routinely considered in patients with advanced AVB.

While used for European populations, the original SILC can be modified to a seven-parameter score, including the following components: (1) age lower than 75 years; (2) risky outdoor activity and living in the countryside; (3) tick bite; (4) constitutional symptoms of Lyme disease; (5) erythema migrans; (6) male sex and (7) fluctuating atrioventricular block.

## Figures and Tables

**Table 1:** Patients’ assignment to groups according to serology results.

ELISA		Western Blott		Interpretation		Study Group
IgM	IgG	IgM	IgG			
(−)	(−)	n.p.	n.p.	Negative *Borrelia burgdorferi* serology (NBS)		Non-Lyme Carditis
(−) *	(−)	(−)	(−)			
(+)	(−)	(−)	(−)			
(−) *	(+)	(−)	(−)			
(+)	(+)	(−)	(−)			
(−) *	(+)	(−)	(+)	Positive *Borrelia burgdorferi* serology	Possible previous *Borrelia* exposure (pBPE)	
(+)	(+)	(−)	(+)			
(+)	(−)	(+)	(−)		Lyme Carditis (LC)	Lyme Carditis
(+)	(+)	(+)	(+)			

Abbreviations: IgM/IgG—immunoglobulin class M/G. (−)—negative; (−) *—negative or borderline; (+)—positive; n.p.—not performed.

**Table 2:** Modifications of SILC scores.

SILC Variant	Components	Score Range	No of Score Components	Modification from the Original SILC
SILC	A^50^, OA/EA, TB, CS, EM, S_m_	0–12	6	None (all patients had EA point added)
SILC_1_	A^50^, **OA/EA^c^**, TB, CS, EM, S_m_	0–12	6	Countryside as Endemic Area parameter
SILC_2_	**A^75^**, OA/EA, TB, CS, EM, S_m_	0–12	6	Age parameter changed to age < 75 years old
SILC_3_	**A^75^**, **OA/EA^c^**, TB, CS, EM, S_m_	0–12	6	Age parameter changed to age < 75 years old and Countryside as Endemic Area parameter
SILC_4_	**A^75^**, **OA/EA^c^**, TB, CS, EM, S_m,_**fAVB**	0–13	7	SILC_4_ enriched with fAVB parameter

Abbreviations: SILC—The Suspicious Index in Lyme Carditis; A^50^—Age < 50; A^75^—Age < 75; OA/EA—outdoor activity/endemic area; TB—tick-bite; EM—erythema migrans; S_m_—male sex; CS—constitutional symptoms; EA^c^—countryside as endemic area; fAVB—fluctuating atrioventricular block; modified parameters given in **bold**.

**Table 3:** Clinical characteristics.

	Lyme Carditis N = 16	No Lyme Carditis N = 114	*p*-Value
Age	61.9 ± 17.0	68.2 ± 17.6	0.08
Sex, male	11 (68.8%)	69 (60.5%)	0.53
Arterial Hypertension	12 (75.0%)	72 (63.2%)	0.22
Diabetes	4 (25.0%)	36 (31.5%)	0.25
Ischemic Heart Disease	4 (25.0%)	30 (26.3%)	0.85
Atrial Fibrillation	3 (18.3%)	20 (17.5%)	0.81
COPD	0 (0%)	10 (8.8%)	0.46
Dyslipidaemia	6 (37.5%)	38 (33.3%)	0.96
Heart Failure	2 (12.5%)	21 (18.4%)	0.81
Ejection Fraction, %	56.2 ± 8.7	59.1 ± 12.1	0.40
White Blood Count, /mcl	8.0 ± 2.4	8.1 ± 3.0	0.97
C-reactive protein, mcg/L	5.5 ± 6.8	7.8 ± 14.9	0.56
*Bradyarhythmia symptoms*			
None	1 (6.3%)	17 (14.9%)	0.17
Effort Intolerance	1 (6.3%)	3 (2.6%)	
Presyncope	10 (62.5%)	52 (45.6%)	
Syncope	4 (25.0%)	42 (36.8%)	
*The most severe AVB recorded*			
Complete Heart Block	6 (37.5%)	45 (39.4%)	0.82
AVB Mobitz type	8 (59.0%)	52 (45.6%)	
AVB Wenckebach type	2 (12.5%)	9 (7.9%)	
Trifascicular block	0 (0%)	7 (6.1%)	
1° AVB	0 (0%)	1 (0.9%)	
*Type of the bradyarhythmia*			
Sinus bradycardia	4 (25.0%)	21 (18.4%)	0.09
Fluctuating AVB	12 (75.0%)	56 (49.1%)	0.04

Abbreviations: COPD—chronic obstructive pulmonary disease; AVB—atrioventricular block.

**Table 4:** Parameters connected with Lyme carditis (SILC score).

	Lyme Carditis N = 16	No Lyme Carditis N = 114	*p*-Value
*SILC components*			
Tick bite	3 (18.5%)	7 (6.1%)	0.11
Age < 50	4 (25.0%)	18 (15.8%)	0.26
Male sex	11 (68.8%)	69 (60.5%)	0.53
Erythema migrans	1 (6.3%)	1 (0.9%)	0.23
Constitutional Symptoms *	2 (12.5%)	1 (0.9%)	0.04
Risky Outdoor Activity	1 (12.5%)	6 (5.3%)	0.61
Endemic Area	16 (100.0%)	114 (100.0%)	1.00
*Modified SILC parameters*			
Age < 75	13 (81.7%)	67 (58.8%)	0.07
Countryside as Endemic Area	7 (43.8%)	31 (27.2%)	0.17
Countryside & Risky Outdoor Activity	7 (43.8%)	32 (27.4%)	0.2
*SILC score results*			
SILC	3.00 ± 2.42	2.01 ± 1.12	0.03
SILC categories: Low Intermediate High			0.07
	1 (6.2%) 12 (75.0%) 3 (18.8%)	14 (12.3%) 96 (84.2%) 4 (3.5%)	
SILC_d_ categories: Lower Higher			0.48
	1 (6.2%) 15 (93.8%)	14 (12.3%) 100 (87.7%)	
SILC_1_	2.43 ± 2.34	1.29 ± 1.25	0.01
SILC_2_	3.56 ± 2.31	2.44 ± 1.20	0.008
SILC_3_	3.00 ± 2.22	1.72 ± 1.31	0.003
SILC_4_	3.75 ± 2.41	2.21 ± 1.44	0.001

Abbreviations: SILC—The Suspicious Index in Lyme Carditis; SILC_d_—original SILC-dichotomous categories; SILC_1,2,3,4_—modifications of SILC—see Table 2; * “constitutional symptoms” included arthralgia in all patients and additionally myalgia and neuropathy in 1 patient from LC group.

**Table 5:** Receiver operation characteristic analysis and logistic regression analysis for SILC and its variants.

Score	AUC	*p*-Value_(AUC)_	Cut-Off Value	SE	SP	OR [± 95%CI]	*p*-Value_(OR)_
SILC	0.664	0.019	>2	31.3%	87.7%	3.25 [0.97; 10.86]	0.054
SILC_1_	0.694	0.006	1.5	62.5%	66.7%	3.33 [1.11; 9.96]	0.029
SILC_2_	0.705	0.004	2.5	75.0%	62.3%	4.95 [1.49; 16.53]	0.008
SILC_3_	0.729	0.001	1.5	87.5%	48.3%	6.56 [1.39; 30.47]	0.016
SILC_4_	0.754	<0.001	2.5	87.5%	63.2%	12.00 [2.56; 56.22]	0.0015

Abbreviations: SILC—The Suspicious Index in Lyme Carditis; SILC_1,2,3,4_—modifications—see Table 2; AUC—area under curve; CI—confidence interval; OR—odds ratio, SE—sensitivity, SP—specificity.

## Data Availability

Not applicable.
